# A Case of Small Bowel Arteriovenous Malformation Diagnosed Using Multiphase CT Angiography and Digital Subtraction Angiography

**DOI:** 10.7759/cureus.42644

**Published:** 2023-07-29

**Authors:** Soichiro Okamoto, Yusuke Matsui, Hiroyuki Sakae, Keiichiro Oshima, Takao Hiraki

**Affiliations:** 1 Radiology, Tsuyama Chuo Hospital, Okayama, JPN; 2 Radiology, Faculty of Medicine, Dentistry and Pharmaceutical Sciences, Okayama University, Okayama, JPN; 3 Internal Medicine, Tsuyama Chuo Hospital, Okayama, JPN; 4 Surgery, Tsuyama Chuo Hospital, Okayama, JPN

**Keywords:** digital subtraction angiography (dsa), small bowel arteriovenous malformation, ct angiography, small bowel resection, small bowel bleeding

## Abstract

Small bowel arteriovenous malformation (AVM) is a rare vascular lesion, which should be considered in patients presenting with gastrointestinal bleeding, as it is a high-flow arterial lesion that can cause life-threatening bleeding. Although a primary endoscopic examination is performed in cases of suspected small bowel bleeding, the diagnosis of the causal lesion is sometimes difficult. We are presenting a case of small bowel AVM that could not be diagnosed endoscopically but was successfully detected using multiphase CT images with an appropriate protocol. The AVM diagnosis was confirmed using digital subtraction angiography. An endovascular coil is placed in the draining vein as a surgical resection marker. The AVM was resected successfully without any complications.

## Introduction

Small bowel bleeding constitutes the majority of obscure gastrointestinal bleeding cases [1], for which the primary diagnosis and treatment is done endoscopically. Video capsule endoscopy is considered to be the initial modality for small bowel evaluation of a suspected small bowel bleed, and deep enteroscopy is attempted based on the clinical presentation and findings of VCE [2]. Although deep enteroscopy offers diagnostic advantages over video capsule endoscopy and provides therapeutic options, it requires a high level of technical expertise and has been reported to have a pooled detection rate of 68.1% for all small bowel diseases [3]. Herein, we describe a case of small bowel arteriovenous malformation (AVM) that could not be diagnosed endoscopically but was diagnosed using multiphase CT angiography with an appropriate scan protocol and subsequent digital subtraction angiography.

## Case presentation

**Figure 1:**
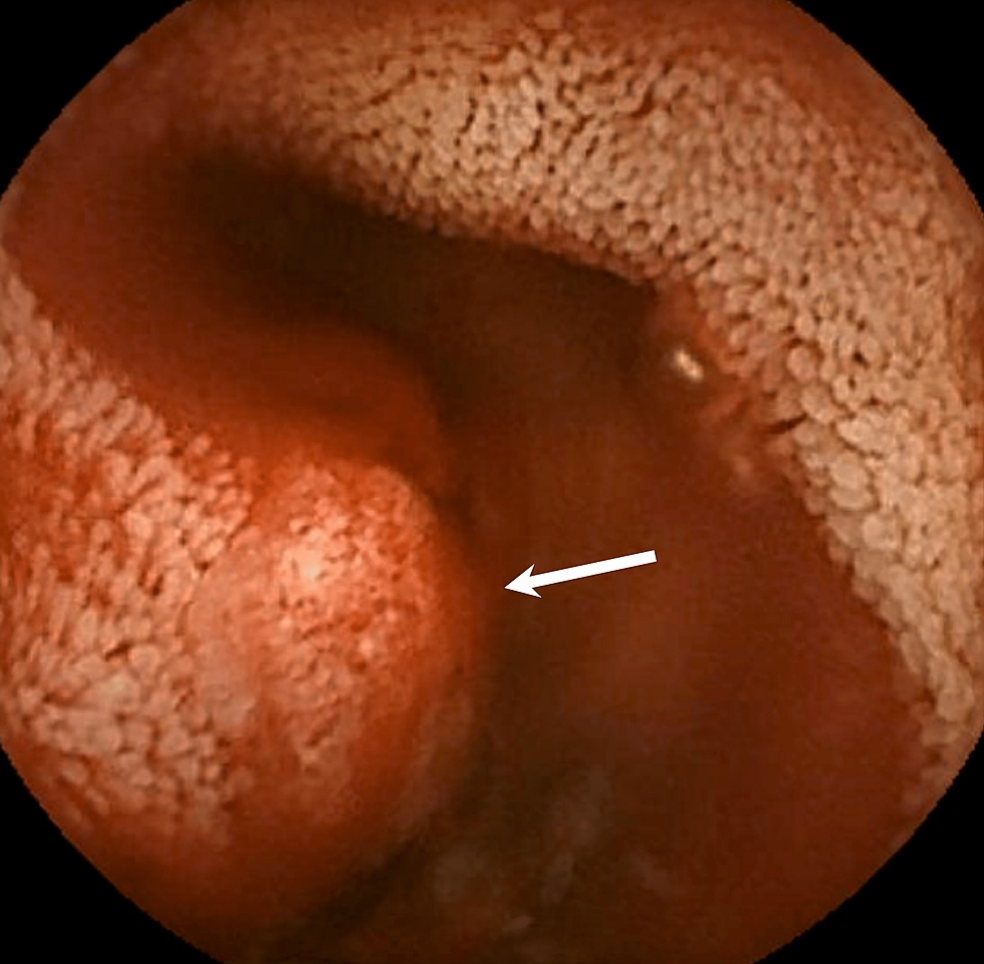
Video capsule endoscopy demonstrates an erythematous protruding subepithelial lesion with bleeding (white arrow) in the mid small bowel.

A 62-year-old man with cirrhosis presented with anemia and melena. He had a history of three endoscopic injection sclerotherapy and ligation for worsening or ruptured esophageal varices within one year. His hemoglobin decreased from 13.3 g/dL to 7.6 g/dL in one year. Upper and lower endoscopies revealed no recurrence of the varices or any other bleeding lesions. Video capsule endoscopy revealed an erythematous, protruding subepithelial lesion in the mid small bowel (Figure 1).

 

**Figure 2:**
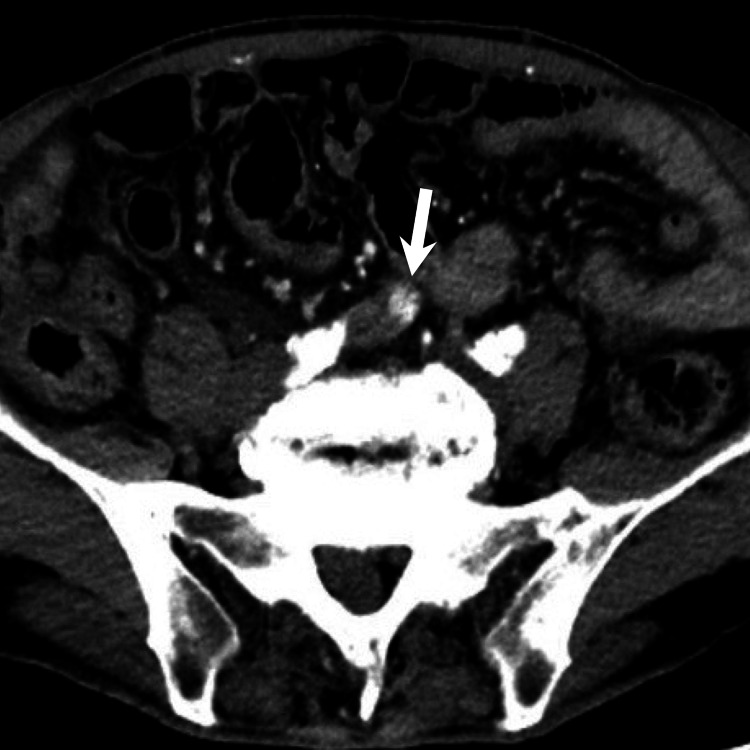
An arterial phase image of multiphase CT angiography reveals an enhanced lesion measuring 1 cm in the small bowel, corresponding to the nidus of the arteriovenous malformation (AVM) (white arrow).

**Figure 3:**
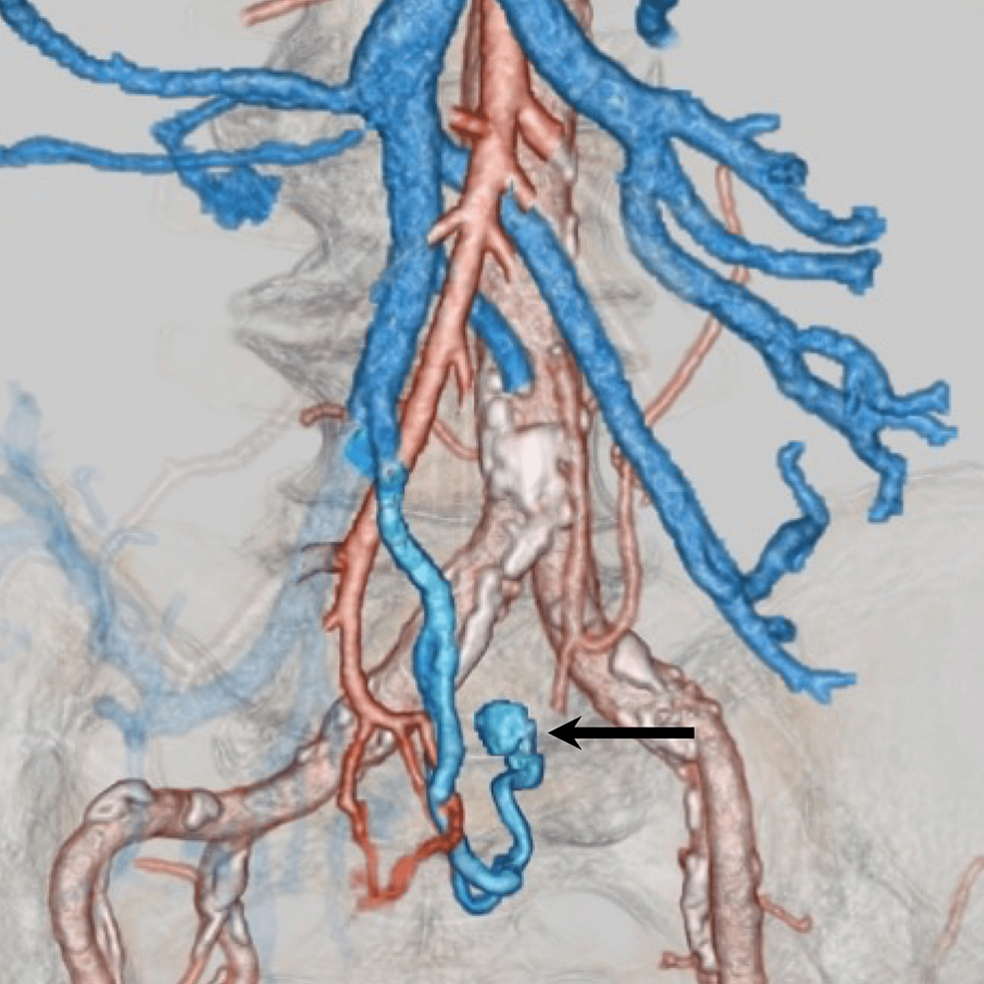
A three-dimensional reconstruction image demonstrates the AVM nidus (black arrow) and a dilated drainage vein. AVM - arteriovenous malformation

**Figure 4:**
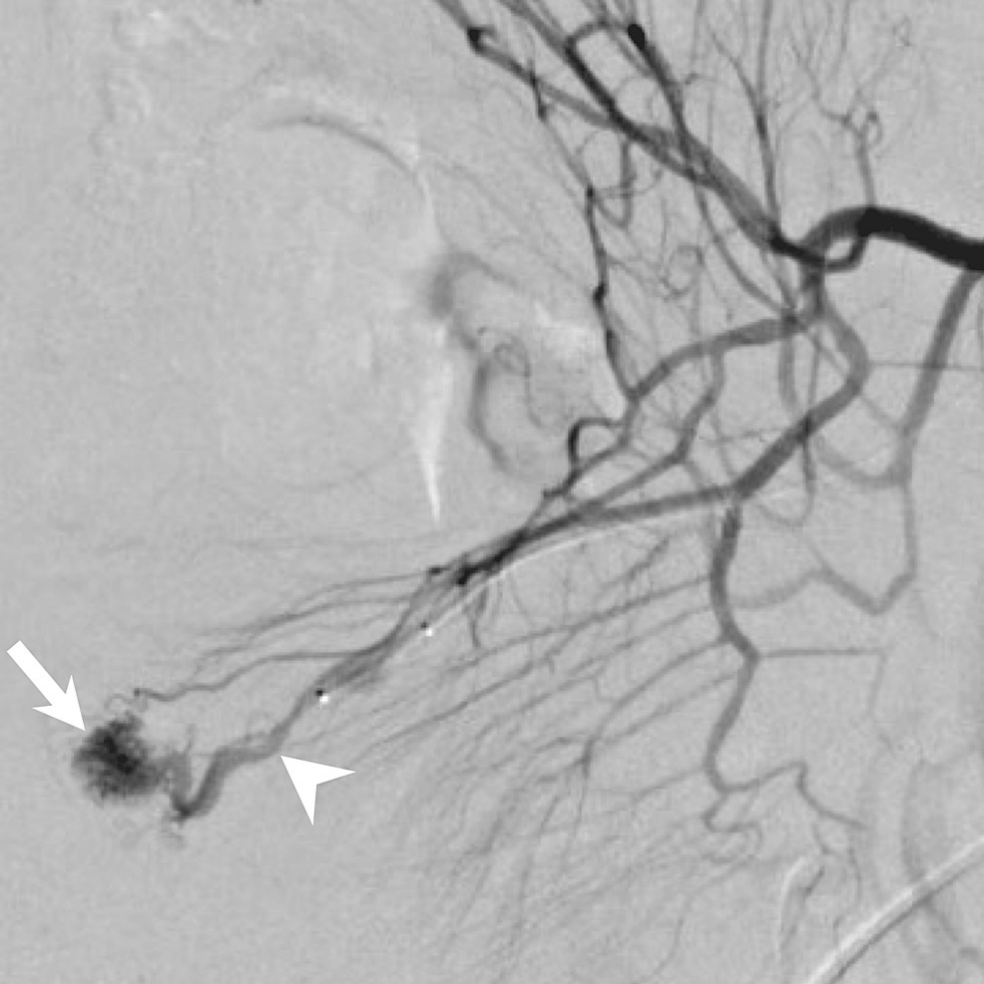
Superior mesenteric arteriography demonstrates multiple feeders, the nidus (white arrow), and the draining vein (white arrowhead), confirming the diagnosis of AVM.

**Figure 5:**
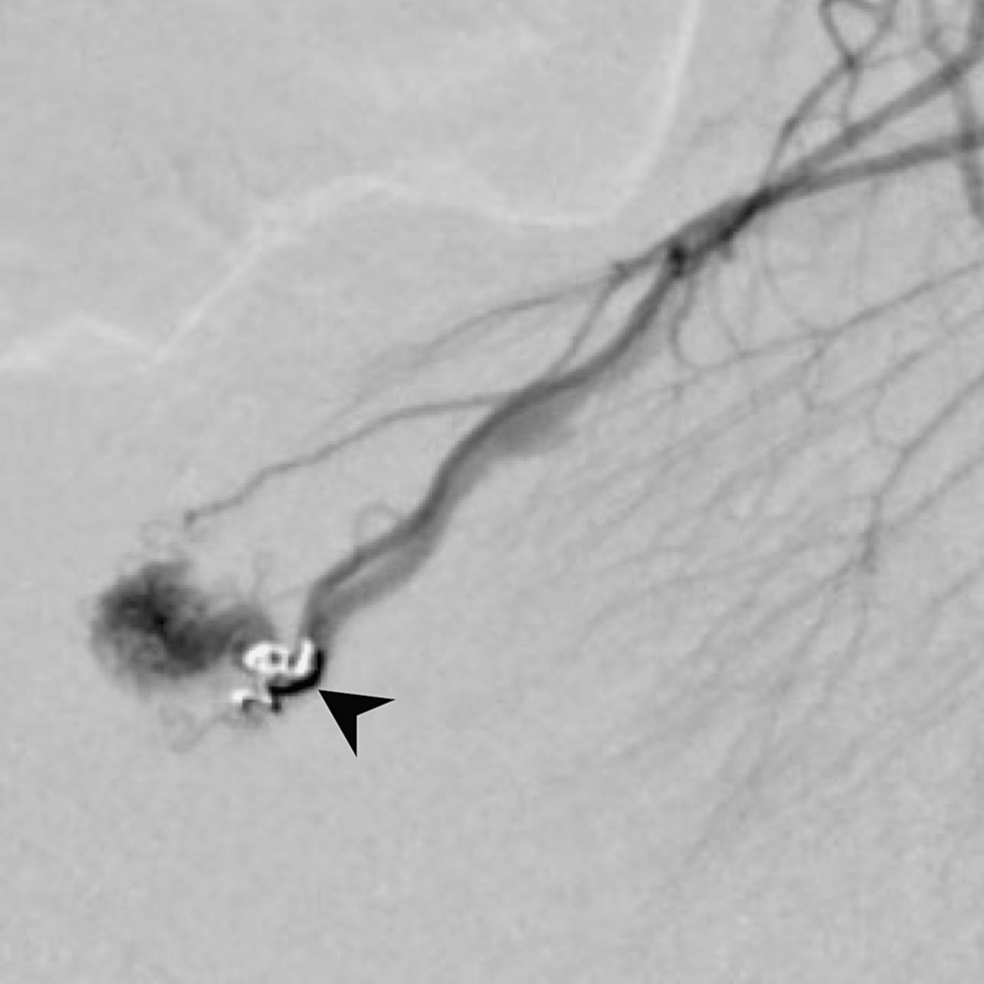
Coils are placed in the draining vein close to the lesion (black arrowhead).

Double-balloon endoscopy was performed for the evaluation and diagnosis. However, it failed to detect the lesion because the endoscope did not reach the lesion through either the oral or the anal route. Subsequently, multiphase CT angiography was performed using a protocol that included the arterial, portal venous, and delayed phases (40, 60, and 210 s after injecting 100 ml of contrast material at a rate of 3.6mL/s, respectively). However, a small bowel lesion was not detected during the initial CT examination. Due to the patient's stable condition, conservative treatment with iron supplementation was continued, but his anemia did not improve, with a hemoglobin of 7.4 g/dL after four months. Thus, the multiphase CT angiography was repeated for a more detailed evaluation using the bolus-tracking method, with the region of interest set within the abdominal aorta at the level of the celiac artery, using a trigger attenuation threshold of 150 HU. The arterial-phase images were obtained after an additional trigger delay of 10 s. The portal venous- and delayed-phase images were obtained 20 and 170 s after the arterial phase, respectively. The arterial phase images revealed an abnormally enhanced lesion measuring 1 cm in size in the small bowel. The lesion was connected directly to a dilated ileal vein, suggesting the presence of a vascular lesion (Figures 2, 3).

,

The patient was hemodynamically stable, but persistent bleeding was suspected, and invasive treatment was considered necessary. Digital subtraction angiography, including percutaneous transhepatic portography and superior mesenteric arteriography, was therefore performed to confirm the diagnosis and to embolize the lesions. The portography and balloon-occluded retrograde angiography via the draining vein did not reveal any small bowel lesions. Subsequently, the superior mesenteric arteriography demonstrated multiple tiny feeding ileal arteries supplying the lesion and early venous drainage to the ileal vein, confirming the diagnosis of an AVM (Figure 4). Surgical resection was preferred considering the challenges associated with selective embolization, such as the necessity of embolizing multiple fine feeders, the risk of intestinal ischemia by embolization, and rebleeding due to inadequate/incomplete embolization. Two microcoils (Target XL 360 soft; 2 mm×3 cm; Stryker, Kalamazoo, USA) were placed in the draining vein close to the lesion as markers for the resection (Figures 4, 5).

**Figure 6:**
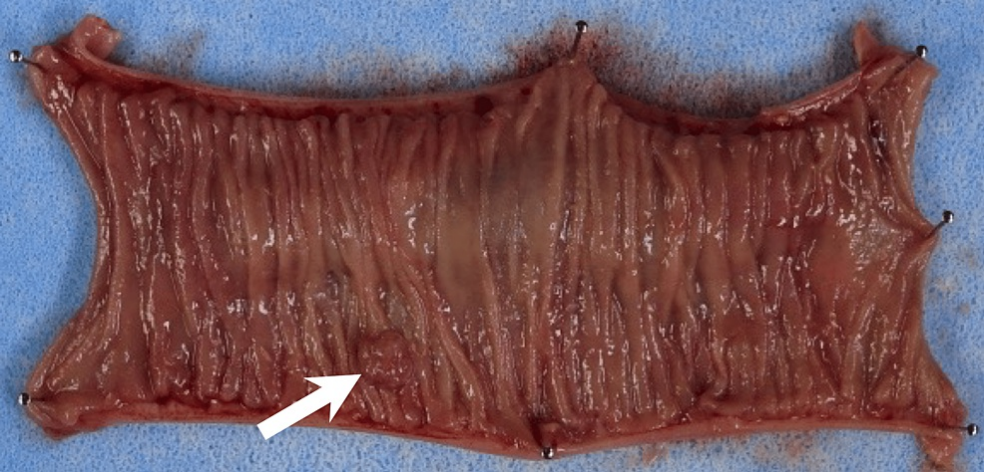
A macroscopic image of the resected ileum shows a protruding lesion (white arrow), diagnosed histopathologically as an ileal arteriovenous malformation.

One week later, an open partial ileal resection and manual anastomosis were successfully performed. Intraoperative fluoroscopy demonstrated the coil clearly, aiding in the localization of the lesion and the determination of the resection line. A histopathological examination confirmed the diagnosis of an ileal AVM (Figure 6). The anemia improved after surgery, and the patient was discharged without complications.

## Discussion

Small bowel vascular lesions are the most common causes of obscure gastrointestinal bleeding [4]. They are classified into three types: angioectasias, arterial lesions, and venous lesions [5]. Arterial lesions, such as AVM or Dieulafoy lesions, should be the prime consideration in cases of acute small bowel bleeding because they can cause life-threatening conditions [1]. Evaluating the presence of a high-flow arterial component in vascular lesions is crucial for selecting an optimal treatment approach and saving a patient’s life [4]. Pulsation during the endoscopic observation is indicative of an arterial component [4]. Furthermore, multiphase CT is useful for the diagnosis of arterial lesions; high-flow components are enhanced prominently during the arterial phase and become less apparent in the venous and delayed phases [1,5]. CT angiography based on the multiphase CT technique has been reported to be useful in diagnosing acute gastrointestinal bleeding, with a pooled sensitivity of 89% and a specificity of 85% throughout the gastrointestinal tract [6]. During the multiphase CT, the acquisition of appropriate arterial-phase images is particularly important to demonstrate AVMs clearly. In the present case, the arterial phase in the initial CT examination, acquired with a fixed delay time of 40 s after the contrast injection, was considered to be too late to demonstrate an AVM. The bolus-tracking method was used in the second CT examination, enabling the acquisition of the arterial phase at the optimal time and the successful demonstration of the AVM.

AVMs exhibit characteristic angiographic features, including dilated arteries, a nidus, and an early venous return [7]. Therefore, angiography may be the final diagnostic modality for AVM. Most small bowel AVMs require surgical resection because of their propensity for re-bleeding [4]. However, identifying AVM during surgery is sometimes challenging, particularly when the lesions are small. Endovascular coil placement has been reported to be useful for localizing small bowel AVMs [8]. This procedure can be performed after diagnostic angiography when surgical resection is indicated, as in the present case.

## Conclusions

We encountered a case of a small bowel AVM that caused bleeding and could not be detected in double-balloon endoscopy. Repeated multiphase CT angiography demonstrated the lesion successfully, and the subsequent digital subtraction angiography confirmed the diagnosis of AVM. Thus, multiphase CT angiography using an appropriate protocol can serve as a salvage diagnostic examination method. Endovascular coil placement following diagnostic angiography is useful for localizing lesions during surgical resection.
